# Development of a serum-free co-culture of human intestinal epithelium cell-lines (Caco-2/HT29-5M21)

**DOI:** 10.1186/1471-2121-7-20

**Published:** 2006-05-02

**Authors:** Géraldine Nollevaux, Christelle Devillé, Benaïssa El Moualij, Willy Zorzi, Patricia Deloyer, Yves-Jacques Schneider, Olivier Peulen, Guy Dandrifosse

**Affiliations:** 1 diGESD (Study Group of Digestive System), Center of Immunology, Institute of Pathology, University of Liege, 4000 Liege, Belgium; 2 Department of human histology, Center of Immunology, CRPP, Institute of Pathology, University of Liege CHU, 4000 Liege, Belgium; 3 Department of Cellular Biochemistry, Catholic University of Louvain, 1348 Louvain-la-Neuve, Belgium

## Abstract

**Background:**

The absorptive and goblet cells are the main cellular types encountered in the intestine epithelium. The cell lineage Caco-2 is a model commonly used to reproduce the features of the bowel epithelium. However, there is a strong debate regarding the value of Caco-2 cell culture to mimick *in vivo *situation. Indeed, some authors report in Caco-2 a low paracellular permeability and an ease of access of highly diffusible small molecules to the microvilli, due to an almost complete lack of mucus. The HT29-5M21 intestinal cell lineage is a mucin-secreting cellular population. A co-culture system carried out in a serum-free medium and comprising both Caco-2 and HT29-5M21 cells was developed. The systematic use of a co-culture system requires the characterization of the monolayer under a given experimental procedure.

**Results:**

In this study, we investigated the activity and localization of the alkaline phosphatase and the expression of *IAP *and *MUC5AC *genes to determine a correlation between these markers and the cellular composition of a differentiated monolayer obtained from a mixture of Caco-2 and HT29-5M21 cells. We observed that the culture conditions used (serum-free medium) did not change the phenotype of each cell type, and produced a reproducible model. The alkaline phosphatase expression characterizing Caco-2 cells was influenced by the presence of HT29-5M21 cells.

**Conclusion:**

The culture formed by 75% Caco-2 and 25% HT29-5M21 produce a monolayer containing the two main cell types of human intestinal epithelium and characterized by a reduced permeability to macromolecules.

## Background

Among human intestinal diseases, intestinal inflammatory disorders, such as food allergies and Crohn's disease, are characterized by an increase in intestinal permeability [1-4]. The *in vitro *study of these pathologies as well as of permeability modulators requires the use of a cell model representing as closely as possible the physiological conditions.

The small intestinal epithelium is the main barrier preventing the molecules from the lumen (e.g. food, toxins) to reach the blood compartment [5]. This epithelium is composed of several cell types: enterocyte, goblet cell, paneth cell, endocrine cell and stem cell [6,7]. The absorptive and goblet cells are the two main cellular types in the intestinal epithelium. The apical side of the enterocytes is characterized by a brush border which contains several enzymes and which increases the surface for nutrient absorption. The goblet cells secrete a mucus [8,9], which covers the apical membrane of intestinal cells and limits molecule absorption [10,11]. The cellular phenotypes in the epithelium are influenced by cell-cell and cell-matrix interactions and are defined by the expression of specific genes [12].

*In vitro *models are increasingly developed to study drug and nutrient transport across the intestinal epithelium [13-16]. The intestinal cell lineage Caco-2 is the most commonly used cell model [17,18]. However, some authors report in Caco-2 a low paracellular permeability [14,16,19] and an ease of access of highly diffusible small molecules to the microvilli, due to an almost complete lack of mucus [16]. Caco-2 cells were obtained from human colon adenocarcinoma [20]. It differentiates spontaneously when it grows to confluence [21]. The alkaline phosphatase (EC 3.1.3.1.), coded by the *IAP *gene, is an enzyme widely used as a marker of differentiation in the Caco-2 cell type [22,23]. The parental HT29 cell line was obtained from human colorectal cancer [24]. The HT29-5M21 cell line results from the isolation of HT29 cells adapted to methotrexate (MTX, 10^-5 ^M) [25,26]. The differentiation of goblet cells is characterized by the secretion of several mucins [27]. The *MUC5AC *mucin gene, usually expressed in the stomach, accounts for the major expressed mucin gene in HT29-MTX [28,29].

The aim of our study is the development of a serum-free co-culture of human intestinal epithelium cell lines. This reproducible co-culture will form an epithelial monolayer exhibiting the two main cellular types encountered in the human intestinal epithelium. The systematic use of co-culture systems requires the characterization of the monolayer under a given experimental procedure. In this study, we investigated the activity and localization of alkaline phosphatase and the expression of *IAP *and *MUC5AC *genes to determine a correlation between these markers and the cellular composition of a differentiated monolayer obtained from a mixture of Caco-2 and HT29-5M21 intestinal cells.

## Results

### Co-culture characterization

**Figure 1:**
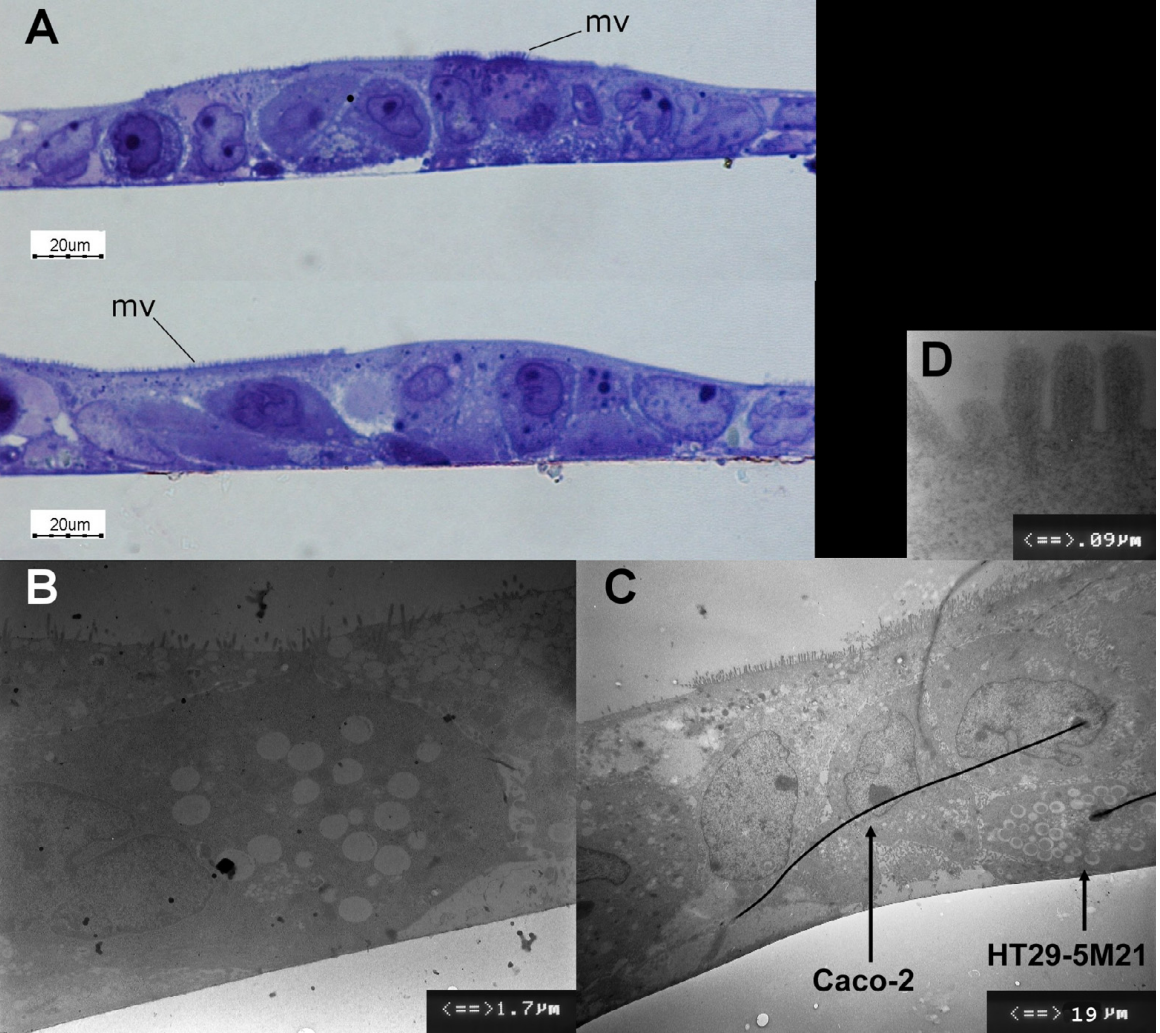
Semi-thin cross sections (toluidine blue staining) (A) and electronic microscopy ultra-thin sections (B-D) of Caco-2/HT29-5M21 co-culture monolayer (seeding ratio 3:1). Mv: microvilli.

**Figure 2:**
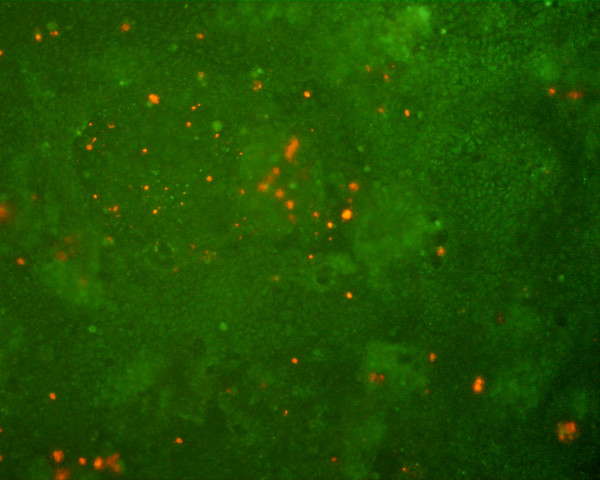
Fluorescent dye exclusion in Caco-2/HT29-5M21 co-culture monolayer (seeding ratio 3:1) 21 days after the confluence.

Microscopy analysis of the Caco-2/HT29-5M21 (seeding ratio 3:1) co-cultures showed a monolayer of differentiated cells with microvilli (Fig. 1). Monolayer formed domes which have been shown to exhibit signs of enterocytic differentiation and transport properties. Viability of the Caco-2/HT29-5M21 (seeding ratio 3:1) co-cultures was assayed by fluorescent dye exclusion (Fig. 2). Living cells were observed with a green fluorescent nuclei. At the end of the culture (21 days after confluence) few cells were observed with an orange fluorescent nuclei/cytoplasm.

### Permeability properties

**Figure 3:**
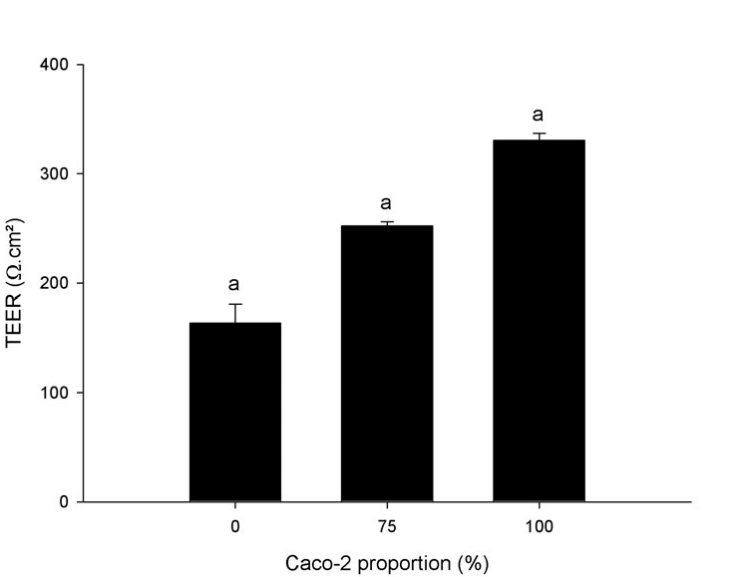
Transepithelial Electrical resistance of Caco-2/HT29-5M21 co-culture monolayer 21 days after the confluence. Results are expressed as Ω.cm^2^. Groups sharing the same letter are significantly different (^a ^p < 0.001, N ≥ 4).

**Figure 4:**
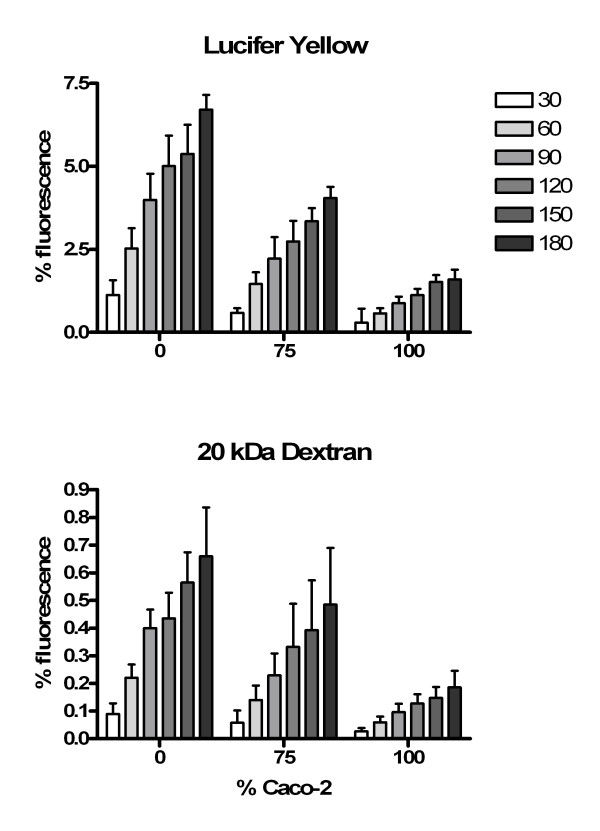
Lucifer yellow (452 Da) and dextran (20 kDa) fluorescence in the basolateral pole compartment of Caco-2/HT29-5M21 co-culture monolayer 21 days after the confluence. Basolateral pole compartment samples were analysed 30 to 180 minutes after permeability tracer addition into the apical compartment. Results are expressed as % of total fluorescence.

Transepithelial electrical resistance (TEER) measurement was assayed 21 days after confluence on Caco-2/HT29-5M21 co-cultures at different seeding ratio (Fig. 3). Results showed a decrease of the TEER with the decrease of the Caco-2 proportion. Monolayer permeability to lucifer yellow and to 20 kDa fluorescent-dextran was assayed 21 days after confluence on Caco-2/HT29-5M21 co-cultures at different seeding ratio (Fig. 4). Permeability of Caco-2/HT29-5M21 co-cultures was 10-fold higher to Lucifer yellow than to 20 kDa-dextran. Results showed a decrease of the permeability to these molecules with the increase of the Caco-2 proportion in the co-culture.

### Enzyme activity and localization

**Figure 5:**
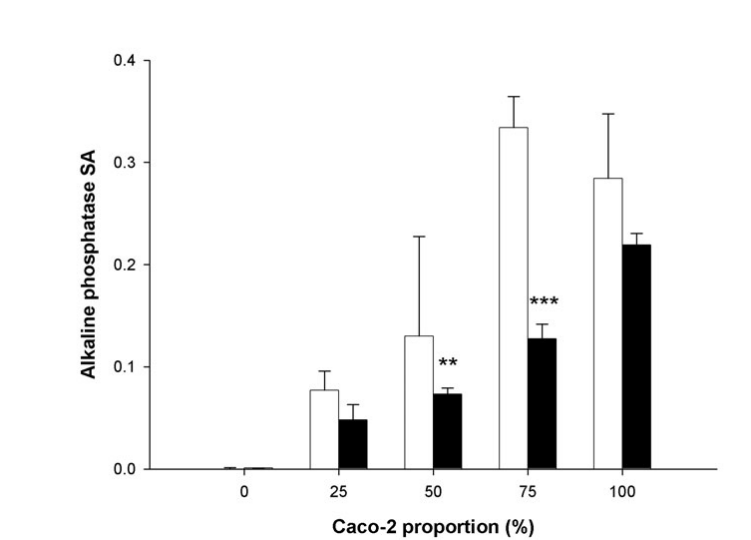
Alkaline phosphatase specific activity measured in Caco-2/HT29-5M21 co-cultures (open bars) and in mixtures (black bars) of these cells in the proportions 3:1, 2:2, 1:3. Results are expressed as specific activity (SA, μmol/min.mg protein). **p < 0.01, ***p < 0.001 (N = 3).

**Figure 6:**
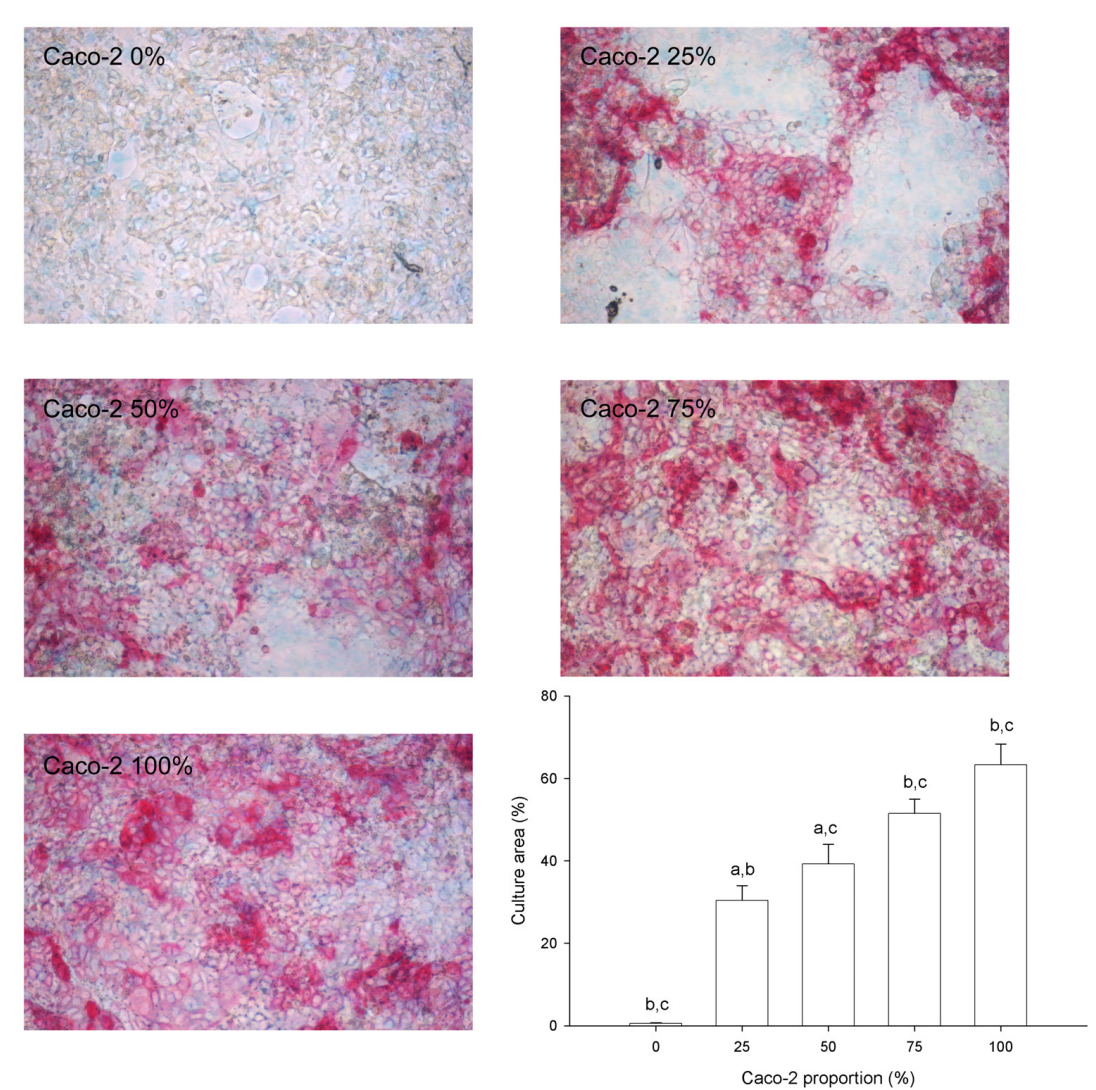
Localization of alkaline phosphatase activity (in red) by cytochemistry in a co-culture of Caco-2 and HT29-5M21. Groups sharing the same letter are significantly different (^a ^p < 0.01; ^b,c ^p < 0.001, N = 4).

In order to study the relationship between enzyme activity and the cellular composition of the monolayer, experiments concerning the specific activity of alkaline phosphatase and enzyme localization were conducted in cell cultures. We used Caco-2 cells, HT29-5M21 cells and co-cultures of these two cell types (seeding ratios 1:3, 2:2, 3:1). The alkaline phosphatase specific activity (SA) was detected in the Caco-2 cultures, whereas no activity was detected in the HT29-5M21 cultures. In the co-cultures and in the cell homogenate mixtures, the SA of alkaline phosphatase increased with the proportion of Caco-2 (p < 0.001 in both cases). The alkaline phosphatase SA was significantly (p < 0.001) greater in the co-cultures than in the mixtures of Caco-2 and HT29-5M21 homogenates (Fig. 5). Comparisons of the pairs showed that this general effect was the result of the 2:2 and 3:1 ratios, where the alkaline phosphatase SA was significantly (respectively p < 0.01 and p < 0.001) greater in the co-cultures than in the mixtures of Caco-2 and HT29-5M21 homogenates. The specific detection of alkaline phosphatase activity was further investigated by cytochemistry. Results showed a significant linear correlation (r = 0.960, p < 0.001) between alkaline phosphatase positive-cells and Caco-2 proportion in the culture. However, Caco-2 culture did not show an alkaline phosphatase localization on the whole culture surface (Fig. 6). A progressive bias towards the Caco-2 proportion and the alkaline phosphatase-positive area resulted from a 2:2 Caco-2/HT29-5M21 seeding ratio.

### Specificity of the primers

**Figure 7:**
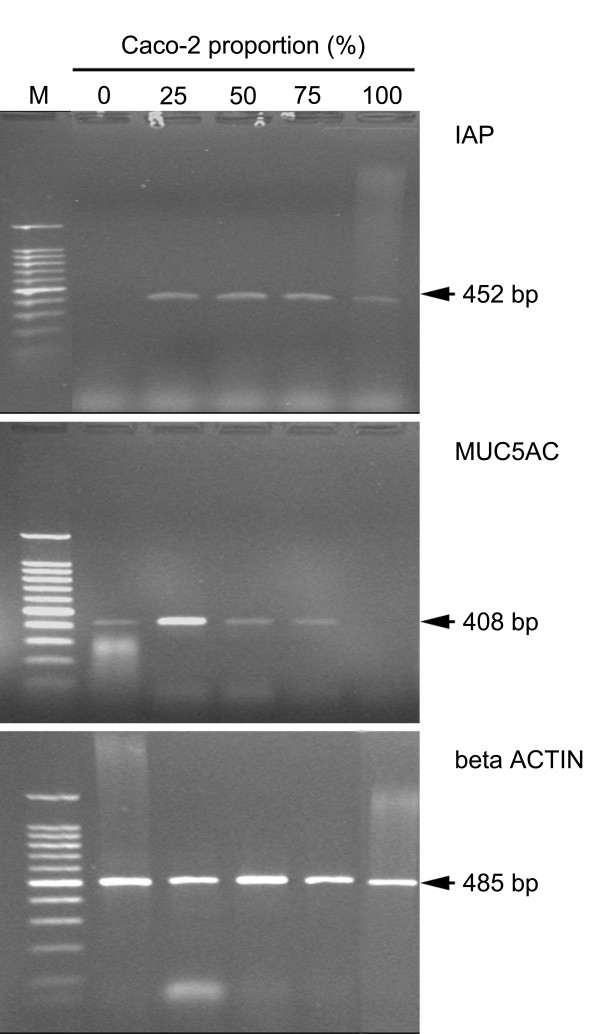
*IAP*, *MUC5AC *and *beta ACTIN *PCR on mRNA isolated from cell cultures. Lanes: M: 100 bp DNA ladder. Analysis was repeated three times.

The specificity of the primer pairs (IAP hu Fo/Ba, MUC5AC Fo/Ba, bACT hu Fo/Ba – see table 1) was tested on Caco-2 and HT29-5M21 cDNA. Caco-2 cDNA gave rise to a 452 bp amplicon with IAP hu Fo/Ba primers and was not amplified with MUC5AC Fo/Ba primers (Fig. 7). HT29-5M21 cDNA gave rise to a 408 bp amplicon with MUC5AC Fo/Ba primers and was not amplified with IAP hu Fo/Ba primers (Fig. 7). In the Caco-2/HT29-5M21 co-culture a 452 or a 408 bp amplicon was detected with, respectively, IAP hu Fo/Ba and MUC5AC Fo/Ba primer pairs (Figs. 7). The bACT hu Fo/Ba primer pairs amplified a 485 bp fragment in Caco-2 and HT29-5M21 cDNA (Fig. 7).

**Table 1:** List of primers used in this study

***Primer sets***	***Sequences 5'→3'***	***Predicted PCR product size (bp)***	***Tm (°C)***	***References***
IAP hu Fo	CCG-CTT-TAA-CCA-GTG-CAA-CA		60	
		452		-
IAP hu Ba	CCC-ATG-AGA-TGG-GTC-ACA-GA		62	

MUC5AC Fo	TGA-TCA-TCC-AGC-AGC-AGG-GT		62	
		408		[25]
MUC5AC Ba	CCG-AGC-TCA-GAG-GAC-ATA-TGG-G		70	

bACT hu Fo	AGA-AAA-TCT-GGC-ACC-ACA-CC		60	
		485		-
bACT hu Ba	GTC-AGG-CAG-CTC-GTA-GCT-CT		64	

### Expression of IAP and MUC5AC genes

The expression level of *IAP *and *MUC5AC *genes was determined by real-time PCR in the Caco-2 and HT29-5M21 co-cultures. After 40 PCR cycles, the fluorescence, corresponding to SYBER Green^® ^binding, was recorded and plotted against the cycle number (data not shown).

Our results showed that the threshold cycle number (Ct) value, corresponding to the amount of *IAP *cDNA, is the same in the Caco-2 culture and in co-cultures at different seeding Caco-2/HT29-5M21 proportions (1:3, 2:2 and 3:1; Fig. 8). With regard to the HT29-5M21 cDNA, no amplification product was detected until a Ct value corresponding to the value of the negative control was obtained without template. The Ct value did not differ significantly with the proportion of Caco-2 cells in the culture. The same results (Ct ~ 27) were obtained for *MUC5AC *(data not shown).

**Figure 8:**
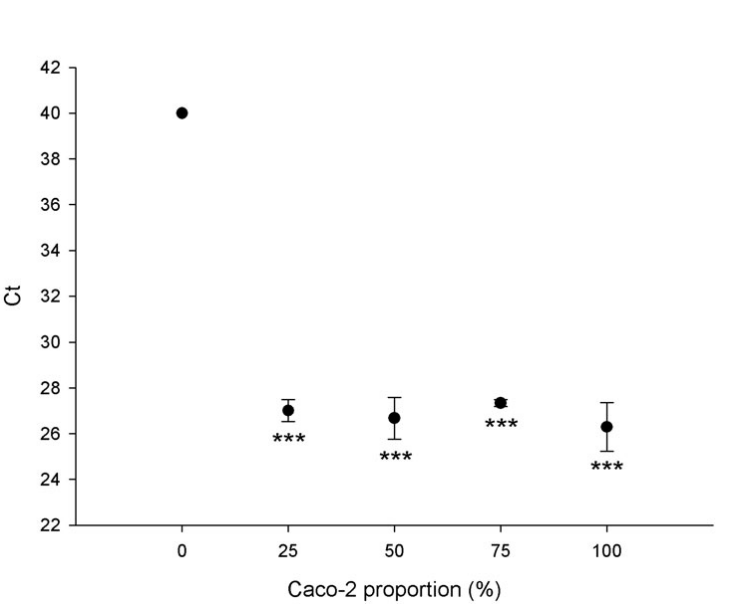
Alkaline phosphatase real-time PCR Ct values against the Caco-2/HT29-5M21 seeding ratio. *** p < 0.001 in comparison with the 0% Caco-2 condition (N ≥ 3).

## Discussion

To understand the cellular events leading to a leaky gut in pathologies such as inflammatory bowel diseases, an *in vitro *approach using cell models is required. The intestinal cell lineage Caco-2 is the cell model most commonly used to reproduce the features of the human small intestine epithelium [17,18]. Nevertheless, the Caco-2 cell model is different in some respects to the human epithelium. For example, it has a low paracellular permeability and allows access of highly diffusible small molecules to the microvilli too easily [14,17,19,30,31], owing to the lack of an adequate mucus layer. As an alternative, therefore, we analyzed the properties of a co-culture of Caco-2 cells and HT29-5M21 cells differentiating spontaneously into mucus-secreting cells. This co-culture system exhibits the two main important cellular types encountered in the human intestinal epithelium. In order to make this procedure reliable and reproducible, we replaced the usual serum-containing culture medium with a serum-free medium supplemented with hormones and amino acids. Although the proportions of both cellular types are determined at culture seeding, they can rapidly modify during culture owing to the ability of each cell line to adhere to and to proliferate on the culture substrate. More, the heterogeneity of Caco-2 cells between 0 and 30 days of confluence [32] can deeply influence the co-culture composition.

A brief characterization of the Caco-2/HT29-5M21 co-culture, initially seeded with a 3:1 ratio, was performed. Results showed a monolayer containing few cells with condensed DNA (ethidium bromide accumulation). The ethidium bromide accumulation may reflect an early proliferative/apoptotic activity and modified nucleic acid expression. Indeed, an increase in DNA content is a feature of actively-proliferating cells that would therefore exhibit higher fluorescence peaks when labeled by ethidium bromide [33]. Moreover, previous studies reported the appearance of an increase in DNA staining by ethidium bromide during the early phases of apoptosis, known as the hyperdiploid peak, [34]. On the other hand, intracellular ethidium bromide fluorescence could also be enhanced by an increase in DNA base pair accessibility associated with the differentiation state [35]. Then, a recent study indeed suggests that cell proliferation and apoptosis can co-exist [36]. In our case we can not speculate between these possibilities. Whatever the solution may be, our results show that our co-culture has a high proportion of living cells.

For this study, we used alkaline phosphatase as an indicator of the cell proportion in co-culture models. The alkaline phosphatase is a membrane-bound enzyme localized in the brush border of enterocytes in the human intestinal epithelium and was used previously by Matsumoto *et al*. [23] to monitor the differentiation of enterocyte-like cells. In order to analyze the correlation between the proportion of the enterocyte-like cells and the alkaline phosphatase in the Caco-2/HT29-5M21 co-cultures, we estimated the enzyme SA. This enzyme activity is increased with the Caco-2 proportion. However, the alkaline phosphatase SA in co-cultures seems to be higher than in cell mixtures with the same proportions (1:3, 2:2 and 3:1). This result suggests a positive interaction between Caco-2 and HT29-5M21 cells. This interaction could lead to an increase of the gene expression, an increase of the translation rate or a decrease of enzyme degradation. This last possibility was already proposed by Matsumoto *et al*. [23] during cell differentiation.

The alkaline phosphatase activity was detected in co-cultures by the cytochemistry method. Our results show that the alkaline phosphatase was restricted to the enterocyte-like cell and could then be used as a potential marker for the monitoring of these cells under several co-culture conditions. The discrepancy between the alkaline phosphatase-positive area and the Caco-2 seeding proportion in culture could be explained by the heterogeneity of the cell lineage [32]. Indeed, this cell line differentiates spontaneously in enterocyte-like cells, but also in other cell types which do not express alkaline phosphatase. Our results underline this heterogeneity, as we observed, by cytochemistry at cellular level, several red-scales (meaning alkaline phosphatase activity) in the same culture. This differentiation seems to be influenced by the Caco-2/HT29-5M21 co-culture and thus by a cell-cell interaction.

In order to collect more data on the proportionate composition of the monolayer, we investigated the expression level of the alkaline phosphatase coding gene and of the *MUC5AC *gene. Though the two cell lineages were both of an adenocarcinoma origin [16,21,24], we showed that the *IAP *and *MUC5AC *gene expressions are restricted, respectively, to Caco-2 cells and HT29-5M21 cells. We showed by real-time PCR that the expression level of the *IAP *and *MUC5AC *genes in the monolayer is independent of the cell proportion and thus of the Caco-2 or HT29-5M21 abundancy. However, the specific activity of the alkaline phosphatase was dependant on the Caco-2 proportion. Consequently, there is a complex control of the gene expression, enzyme translation and degradation regarding the cell-cell interaction.

Monolayer permeability is influenced by the presence of HT29-5M21 cells in the system. This influence is in accordance with an increase of the permeability. Indeed, we showed a decrease of the TEER and an increase of the Lucifer yellow and 20 kDa dextran permeability with the increase of HT29-5M21 proportion in the monolayer at seeding. The cell ratio is established at seeding and is probably not indicative of the situation when permeability is measured. However, HT29-5M21 cells seem to remain in sufficient proportion in the culture to increase cell permeability. The range of molecular masses (Lucifer yellow 452 Da & dextran 20 kDa) used in this study to explore monolayer permeability allow to characterize para-cellular permeability [37,38]. Lucifer yellow is not absorbed by epithelial cell but is able to pass through cell junctions as mannitol does. Dextrans are largely used as macromolecule permeability tracer.

## Conclusion

The phosphatase alkaline, a well-established marker for enterocyte-like cells, could be proposed, in combination with *MUC5AC*, as a new marker of great interest for monitoring the cellular composition of the co-culture. We are currently developing an immuno-labelling system to quantify the level of this protein.

On the basis of the enterocyte/goblet cell ratio observed *in vivo *[39,40], the cell proportion 3:1 allows us to decrease the permeability to macromolecules who becomes closer to the situation observed in a human intestinal epithelium.

Permeability studies, using this co-culture, indicate that Caco-2/HT29-5M21 co-culture is more permissive than Caco-2 cell culture regarding to para-cellular permeability. This characteristic is probably in accordance with the *in vivo *permeability. We can expect to use the model to study some human diseases involving permeability alterations as coeliac disease.

## Methods

### Chemicals

All chemicals were purchased from Sigma-Aldrich (Poole, Dorset, UK) and from VWR (Brussels, Belgium). Cell culture media were obtained from Gibco BRL (Merelbeke, Belgium) and from Sigma-Aldrich. The molecular biology reagents were obtained from Promega (Madison, USA). Lucifer yellow and 20 kDa-dextran were obtained from Sigma-Aldrich.

### Cell lines and culture conditions

The HT29-5M21 cell line, derived from the HT29-MTX 10^-5^M cell line [27], was obtained at passage 9 from Dr. T. Lesuffleur (INSERM U505, Villejuif, France). Caco-2 cells were obtained at passage 22 from Cambrex Bio Science (Verviers, Belgium). These cells were progressively adapted (until passage 30 for HT29-5M21 and passage 40 for Caco-2) to a serum-free medium: the basal defined medium (BDM) [41] supplemented with a mixture of non-essential amino acids and growth factors [42]. Initially, Caco-2 and HT29-5M21 cells were cultured in Dulbecco's Modified Eagle Medium (DMEM) containing glucose (4.15 mg/l) buffered with HEPES (25 mM) containing fetal calf serum (10%) and antibiotics (1%: penicillin [1 U/ml], streptomycin [1 μg/ml] and amphotericin as fungizone^® ^[2.5 ng/ml]). Then, these cells were progressively sub-cultured in BDM containing glucose (4.15 mg/l) supplemented with non-essential amino acids and growth factors [42]. Cells were used at passages 30 (HT29-5M21) and 40 (Caco-2) to avoid dedifferentiation [43]. The cells were seeded at 4 × 10^4 ^cells/cm^2 ^in plastic flasks (Greiner labortechnik SA, Wemmel, Belgium) and cultured at 37°C in a CO_2_-air (5:95) atmosphere (Heraeus EK incubator). The culture medium was changed every day.

### Co-cultures

Five types of culture cells were produced: Caco-2 cells alone, HT29-5M21 cells alone and co-cultures of these cells in various seeding ratios (1:3, 2:2 and 3:1). These cultures (4 × 10^4 ^cells/cm^2 ^per flask) were maintained in BDM supplemented with non-essential amino acids, a mixture of growth factors [42] and antibiotics (see above). The medium was changed every two days. Co-cultures were confluent 7 days after the seeding. The Caco-2 and HT29-5M21 cells were maintained in culture during 21 days of differentiation after confluence. Under these conditions, the maximum expression of intestinal differentiation-specific markers was achieved. For the enterocytes, the differentiation marker was the alkaline phosphatase activity associated with the microvilli [17,30,31]. With regard to the goblet cells, the differentiation marker was the mucin synthesis [44]. Cells were used for mRNA extraction, enzyme assays and localization. Cells in co-culture were briefly characterized by phase contrast microscopy, light microscopy (semi-thin cross sections stained with toluidine blue), electronic microscopy and vital dye (ethidium bromide/acridine orange) exclusion [45].

### Permeability assays

Permeability analyses were performed on cells cultured on 0.4 μm polyethylene terephtalate membrane coated with collagen as described above. Transepithelial electrical resistance measurement was measured in Hank's buffer [46,47]. Lucifer yellow and fluorescent dextran were measured in the baso-lateral pole compartment with a fluorimeter (excitation 430 nm – emission 540 nm).

### Enzyme assays

Enzyme activity assays were performed on cell homogenates. Just before use, the monolayer was washed with PBS buffer (pH 7.2) and 1 ml of water was added. Cells were collected by scraping and were disrupted by ultrasonication (3 times for 20 sec each; ultrasonicer MSE PE818). Enzyme activity was assayed on co-culture homogenates and on Caco-2 and HT29-5M21 homogenate mixtures in 1:3, 2:2 and 3:1 ratios. Alkaline phosphatase activity was assayed according to the method described by Garen and Levinthal [48], with *p*-nitrophenolphosphate as substrate. Results were expressed as milliunit mg^-1 ^of protein (specific activity = SA). One unit was defined as the activity equal to one μmol of substrate hydrolyzed per min at 37°C. Proteins were assayed by the method described by Bradford [49]. Phosphatase alkaline activity was localized on cell culture by cytochemistry using the fast red substrate (naphtol combined to fast red; Roche Diagnostics GmbH, Penzberg, Germany). Labelled cell-culture pictures were analyzed using ImageJ 1.33u software (Wayne Rasband, NIH, USA). Images were converted in 256 greyscale files, then a threshold was defined in order to include the red channel in the selection. The area of the selection was measured as a percentage of the picture surface.

### PCR primers

All the PCR primers used in this study were obtained from Eurogentec (Seraing, Belgium). Three pairs of primers were chosen: IAP hu Fo/Ba was a specific of an alkaline phosphatase gene; MUC5AC Fo/Ba was a specific of the *MUC5AC *mucin gene [29]; and bACT hu Fo/Ba was a specific of the cytosolic *beta actin *coding-gene. The primer3 program [50] was used to design the IAP hu Fo/Ba and bACT hu Fo/Ba primers. The sequences of the primers used in this study are presented in Table I.

### RNA isolation

Total mRNA was isolated using a kit manufactured by Promega (RNagents^® ^total RNA isolation system) from confluent monolayer cells. RNA integrity was checked by electrophoresis in agarose gel (1%) containing ethidium bromide (0.5 μg/ml). RNA concentration was estimated by spectrophotometry at 260 nm.

### Reverse transcription

cDNA was synthesized using 5 μg of DNase-treated mRNA in a reaction mixture containing RT buffer (1x), dNTP (0.6 mM), RNase inhibitor (1 U/μl), OligodT primers (0.2 μM) and RT Mo-MuLV (0.008 U/μl). The mix was incubated for 1 h at 42°C.

### PCR and real-time PCR

The PCR was performed on the cDNA in a final volume of 50 μl. A sample (2 μl) of this cDNA (0.04 μg) was added to the PCR mixture containing Mg^2+ ^free buffer (1x), MgCl_2 _(2.5 mM for IAP hu Fo/Ba, 3 mM for MUC5AC Fo/Ba and 1.5 mM for bACT hu Fo/Ba), dNTP (200 μM), primers (1 μM each) and Taq polymerase (0.1 U). The PCR was carried out with a thermal cycler (Thermolyne, Amplitron^® ^II).

The real-time PCR has been described elsewhere [51]. In our conditions, we used the SYBER^®^Green, which is a double-strand DNA dye that gives rise to a fluorescence when it is intercalated between DNA bases. Real-time PCR was carried out in a final volume of 50 μl containing 25 μl of SYBER^®^Green PCR Master Mix (Applied Biosystems), 2 μl of cDNA (0.04 μg) and 1μM of each primer.

In both procedures, the reaction was performed in duplicate for each cDNA. The PCR conditions were as follows: initial denaturation step at 95°C for 3 min, followed by 40 cycles at 95°C for 1 min, annealing temperature for 1 min (60°C for IAP hu Fo/Ba, 62°C for MUC5AC Fo/Ba and 60°C for bACT hu Fo/Ba) and 72°C for 1 min. A final elongation step was performed for 5 min at 72°C.

The PCR products (10 μl) were separated by electrophoresis in agarose gels (1%) containing ethidium bromide (0.5 μg/ml). The nucleolytic DNA polymerase overlapped the hybridization probe, and the resulting relative increase in the reporter fluorescent dye emission was monitored in real time using a sequence detector (GeneAmp 5700 System, Applied Biosystems). The fluorescence intensity was a function of the cycle number and was determined using the sequence detector software (Applied Biosystems), giving the Ct at which PCR amplification reached the exponential phase. The Ct was plotted against the culture types. The value of the Ct was inversely linearly correlated with the logarithmic value of cDNA quantity.

### Statistics

Results are reported as mean ± standard deviation (SD). With regard to heteroscedasticity, statistical analysis was performed using Newman-Student-Keuls or Kruskal-Wallis ANOVA. A p-value below 0.05 was considered statistically significant.

## Authors' contributions

GN carried out cell culture, molecular biology, biochemistry analysis and experimental design. She was involved in manuscript drafting and revising. CD participated in cell culture and in experimental design. BEM participated in real-time PCR studies and in drafting the manuscript. WZ participated in manuscript revision. PD participated in statistical analysis. OP was involved in morphometry and statistical analysis as well as in drafting and revising manuscript. GD and YJS were the coordinators of the study, participated in the drafting and gave the final approval of the version to be published. All authors read and approved the final manuscript. The authors declare that they have no competing interests.

## References

[B1] Bjarnason I, Macpherson A, Hollander D (1995). Intestinal permeability: an overview. Gastroenterology.

[B2] Lennernas H (1998). Human intestinal permeability. J Pharm Sci.

[B3] DeMeo MT, Mutlu EA, Keshavarzian A, Tobin MC (2002). Intestinal permeation and gastrointestinal disease. J Clin Gastroenterol.

[B4] Farhadi A, Banan A, Fields J, Keshavarzian A (2003). Intestinal barrier: an interface between health and disease. J Gastroenterol Hepatol.

[B5] Baumgart DC, Dignass AU (2002). Intestinal barrier function. Curr Opin Clin Nutr Metab Care.

[B6] Cheng H, Leblond CP (1974). Origin, differentiation and renewal of the four main epithelial cell types in the mouse small intestine. V. Unitarian Theory of the origin of the four epithelial cell types. Am J Anat.

[B7] Potten CS (1998). Stem cells in gastrointestinal epithelium: numbers, characteristics and death. Philos Trans R Soc Lond B Biol Sci.

[B8] Moe H (1953). Mucus-producing goblet cells of the small intestine. Nature.

[B9] Bierring F (1962). Electron microscopic observations on the mucus production in human and rat intestinal goblet cells. Acta Pathol Microbiol Scand.

[B10] Allen JD, Martin GP, Marriott C, Hassan I, Williamson I (1991). Drug transport across a novel mucin secreting cell model: Comparison with the CaCo-2 cell system [abstract]. J Pharm Pharmacol.

[B11] Meaney C, O'Driscoll C (1999). Mucus as a barrier to the permeability of hydrophilic and lipophilic compounds in the absence and presence of sodium taurocholate micellar systems using cell culture models. Eur J Pharm Sci.

[B12] Simon-Assmann P, Kedinger M, De Arcangelis A, Rousseau V, Simo P (1995). Extracellular matrix components in intestinal development. Experientia.

[B13] Wikman-Larhed A, Artursson P (1995). Co-cultures of human intestinal goblet (HT29-H) and absorptive (Caco-2) cells for studies of drug and peptide absorption. Eur J Pharm Sci.

[B14] Walter E, Janich S, Roessler BJ, Hilfinger JM, Amidon GL (1996). HT29-MTX/Caco-2 cocultures as an in vitro model for the intestinal epithelium: in vitro-in vivo correlation with permeability data from rats and humans. J Pharm Sci.

[B15] Hilgendorf C, Spahn-Langguth H, Regardh CG, Lipka E, Amidon GL, Langguth P (2000). Caco-2 versus Caco-2/HT29-MTX co-cultured cell lines: permeabilities via diffusion, inside- and outside-directed carrier-mediated transport. J Pharm Sci.

[B16] Artursson P, Palm K, Luthman K (2001). Caco-2 monolayers in experimental and theoretical predictions of drug transport. Adv Drug Deliv Rev.

[B17] Meunier V, Bourrie M, Berger Y, Fabre G (1995). The human intestinal epithelial cell line Caco-2; pharmacological and pharmacokinetic applications. Cell Biol Toxicol.

[B18] Ingels FM, Augustijns PF (2003). Biological, pharmaceutical, and analytical considerations with respect to the transport media used in the absorption screening system, Caco-2. J Pharm Sci.

[B19] Menon RM, Barr WH (2003). Comparison of ceftibuten transport across Caco-2 cells and rat jejunum mounted on modified Ussing chambers. Biopharm Drug Dispos.

[B20] Pinto M, Robine-Léon S, Appay MD, Kedinger M, Triadou N, Dussaulx E, Lacroix B, Simon-Assmann P, Haffen K, Fogh J, Zweibaum A (1983). Enterocyte-like differentiation and polarization of the human colon carcinoma cell line Caco-2 in culture. Biol Cell.

[B21] Rousset M (1986). The human colon carcinoma cell lines HT-29 and Caco-2: two in vitro models for the study of intestinal differentiation. Biochimie.

[B22] Chantret I, Barbat A, Dussaulx E, Brattain MG, Zweibaum A (1988). Epithelial polarity, villin expression, and enterocytic differentiation of cultured human colon carcinoma cells: a survey of twenty cell lines. Cancer Res.

[B23] Matsumoto H, Erickson RH, Gum JR, Yoshioka M, Gum E, Kim YS (1990). Biosynthesis of alkaline phosphatase during differentiation of the human colon cancer cell line Caco-2. Gastroenterology.

[B24] Fogh J, Trempe G, Fogh J (1975). New human tumor cell lines. Human Tumor Cells in vitro.

[B25] Lesuffleur T, Barbat A, Luccioni C, Beaumatin J, Clair M, Kornowski A, Dussaulx E, Dutrillaux B, Zweibaum A (1991). Dihydrofolate reductase gene amplification-associated shift of differentiation in methotrexate-adapted HT-29 cells. J Cell Biol.

[B26] Wils P, Warnery A, Phung-Ba V, Scherman D (1994). Differentiated intestinal epithelial cell lines as in vitro models for predicting the intestinal absorption of drugs. Cell Biol Toxicol.

[B27] Lesuffleur T, Porchet N, Aubert JP, Swallow D, Gum JR, Kim YS, Real FX, Zweibaum A (1993). Differential expression of the human mucin genes MUC1 to MUC5 in relation to growth and differentiation of different mucus-secreting HT-29 cell subpopulations. J Cell Sci.

[B28] Fergie N, Guo L, Sithole J, Pearson JP, Birchall JP (2003). Influence of prednisolone on the secretion of mucin from the HT29-MTX cell line. Clin Otolaryngol Allied Sci.

[B29] Gouyer V, Wiede A, Buisine MP, Dekeyser S, Moreau O, Lesuffleur T, Hoffmann W, Huet G (2001). Specific secretion of gel-forming mucins and TFF peptides in HT-29 cells of mucin-secreting phenotype. Biochim Biophys Acta.

[B30] Quaroni A, Beaulieu JF (1997). Cell dynamics and differentiation of conditionally immortalized human intestinal epithelial cells. Gastroenterology.

[B31] Artursson P, Borchardt RT (1997). Intestinal drug absorption and metabolism in cell cultures: Caco-2 and beyond. Pharm Res.

[B32] Vachon PH, Beaulieu JF (1992). Transient mosaic patterns of morphological and functional differentiation in the Caco-2 cell line. Gastroenterology.

[B33] Bonaly J, Mestre JC (1981). Flow fluorometric study of DNA content in nonproliferative Euglena gracilis cells and during proliferation. Cytometry.

[B34] Ferlini C, Di CS, Rainaldi G, Malorni W, Samoggia P, Biselli R, Fattorossi A (1996). Flow cytometric analysis of the early phases of apoptosis by cellular and nuclear techniques. Cytometry.

[B35] Darzynkiewicz Z, Traganos F, Kapuscinski J, Staiano-Coico L, Melamed MR (1984). Accessibility of DNA in situ to various fluorochromes: relationship to chromatin changes during erythroid differentiation of Friend leukemia cells. Cytometry.

[B36] Nagy Z, Esiri MM (1998). Neuronal cyclin expression in the hippocampus in temporal lobe epilepsy. Exp Neurol.

[B37] Konsoula R, Barile FA (2005). Correlation of in vitro cytotoxicity with paracellular permeability in Caco-2 cells. Toxicol In Vitro.

[B38] Satsu H, Yokoyama T, Ogawa N, Fujiwara-Hatano Y, Shimizu M (2003). Effect of neuronal PC12 cells on the functional properties of intestinal epithelial Caco-2 cells. Biosci Biotechnol Biochem.

[B39] Cheng H, Leblond CP (1974). Origin, differentiation and renewal of the four main epithelial cell types in the mouse small intestine. I. Columnar cell. Am J Anat.

[B40] Rubio CA, Lindholm J, Rodensjo M (1989). Mapping intestinal metaplasia by histochemistry and morphometry. Pathol Res Pract.

[B41] Schneider YJ (1989). Optimisation of hybridoma cell growth and monoclonal antibody secretion in a chemically defined, serum- and protein-free culture medium. J Immunol Methods.

[B42] Halleux C, Schneider YJ (1991). Iron absorption by intestinal epithelial cells: 1. CaCo2 cells cultivated in serum-free medium, on polyethyleneterephthalate microporous membranes, as an in vitro model. In Vitro Cell Dev Biol.

[B43] Yu H, Cook TJ, Sinko PJ (1997). Evidence for diminished functional expression of intestinal transporters in Caco-2 cell monolayers at high passages. Pharm Res.

[B44] Hennebicq-Reig S, Lesuffleur T, Capon C, De BC, Kim I, Moreau O, Richet C, Hemon B, Recchi MA, Maes E, Aubert JP, Real FX, Zweibaum A, Delannoy P, Degand P, Huet G (1998). Permanent exposure of mucin-secreting HT-29 cells to benzyl-N-acetyl-alpha-D-galactosaminide induces abnormal O-glycosylation of mucins and inhibits constitutive and stimulated MUC5AC secretion. Biochem J.

[B45] Foglieni C, Meoni C, Davalli AM (2001). Fluorescent dyes for cell viability: an application on prefixed conditions. Histochem Cell Biol.

[B46] Lu S, Gough AW, Bobrowski WF, Stewart BH (1996). Transport properties are not altered across Caco-2 cells with heightened TEER despite underlying physiological and ultrastructural changes. J Pharm Sci.

[B47] Yamashita S, Furubayashi T, Kataoka M, Sakane T, Sezaki H, Tokuda H (2000). Optimized conditions for prediction of intestinal drug permeability using Caco-2 cells. Eur J Pharm Sci.

[B48] Garen A, Levinthal C (1960). A fine-structure genetic and chemical study of the enzyme alkaline phosphatase of E. coli. I. Purification and characterization of alkaline phosphatase. Biochim Biophys Acta.

[B49] Bradford MM (1976). A rapid and sensitive method for the quantitation of microgram quantities of protein utilizing the principle of protein-dye binding. Anal Biochem.

[B50] Rozen S, Skaletsky H (2000). Primer3 on the WWW for general users and for biologist programmers. Methods Mol Biol.

[B51] Latil A, Vidaud D, Valeri A, Fournier G, Vidaud M, Lidereau R, Cussenot O, Biache I (2000). htert expression correlates with MYC over-expression in human prostate cancer. Int J Cancer.

